# Protective Effect of Astaxanthin on Blue Light Light-Emitting Diode-Induced Retinal Cell Damage via Free Radical Scavenging and Activation of PI3K/Akt/Nrf2 Pathway in 661W Cell Model

**DOI:** 10.3390/md18080387

**Published:** 2020-07-25

**Authors:** Chao-Wen Lin, Chung-May Yang, Chang-Hao Yang

**Affiliations:** 1 Department of Ophthalmology, National Taiwan University Hospital, Taipei 100, Taiwan; b91401108@ntu.edu.tw (C.-W.L.); chungmay@ntu.edu.tw (C.-M.Y.); 2 Graduate Institute of Clinical Medicine, College of Medicine, National Taiwan University, Taipei 100, Taiwan; 3 College of Medicine, National Taiwan University, Taipei 100, Taiwan

**Keywords:** astaxanthin, blue light, light-emitting diode (LED), photoreceptor, reactive oxygen species (ROS), photo-injury, PI3K, Akt, Nrf2

## Abstract

Light-emitting diodes (LEDs) are widely used and energy-efficient light sources in modern life that emit higher levels of short-wavelength blue light. Excessive blue light exposure may damage the photoreceptor cells in our eyes. Astaxanthin, a xanthophyll that is abundantly available in seafood, is a potent free radical scavenger and anti-inflammatory agent. We used a 661W photoreceptor cell line to investigate the protective effect of astaxanthin on blue light LED-induced retinal injury. The cells were treated with various concentrations of astaxanthin and then exposed to blue light LED. Our results showed that pretreatment with astaxanthin inhibited blue light LED-induced cell apoptosis and prevented cell death. Moreover, the protective effect was concentration dependent. Astaxanthin suppressed the production of reactive oxygen species and oxidative stress biomarkers and diminished mitochondrial damage induced by blue light exposure. Western blot analysis confirmed that astaxanthin activated the PI3K/Akt pathway, induced the nuclear translocation of Nrf2, and increased the expression of phase II antioxidant enzymes. The expression of antioxidant enzymes and the suppression of apoptosis-related proteins eventually protected the 661W cells against blue light LED-induced cell damage. Thus, our results demonstrated that astaxanthin exerted a dose-dependent protective effect on photoreceptor cells against damage mediated by blue light LED exposure.

## 1. Introduction

Artificial lighting is indispensable in our daily life. Light-emitting diodes (LEDs) are energy-efficient light sources that are widely used in consumer electronics, such as smartphones, digital displays, and computer screens. However, LEDs emit higher levels of blue light compared to conventional light sources. Blue light has a wavelength between 400–495 nm, which can penetrate the lens to reach the retina and induce greater retinal damage compared to light sources with longer wavelengths [1]. The main sites for light absorption in the eye are retinal photoreceptors and retinal pigment epithelial (RPE) cells [2]. Previous studies have demonstrated LED-induced photoreceptor and RPE cell damage [3,4]. The association between blue light exposure and age-related macular degeneration (AMD) was also disclosed in epidemiological studies [5].

Oxidative stress and inflammatory reactions play major roles in the mechanism of blue light-induced retinal injury [6,7,8]. AMD is the leading cause of blindness worldwide [9]. Although intravitreal injections of anti-vascular endothelial growth factor are the standard treatment for AMD [10], their effectiveness remains limited. Nutritional supplements for the improvement of eye health have received considerable research attention. Sulforaphane, which is abundant in cruciferous vegetables, exerts anti-oxidative effects and protects RPE cells from photo-oxidative damage [11]. Our research group previously demonstrated the protective effect of chitosan oligosaccharides on blue light LED-induced RPE cell damage [12]. Bilberry and lingonberry have also been proven to protect retinal photoreceptor cells from blue light LED-induced damage [13].

Astaxanthin is a keto-carotenoid [14]. It belongs to xanthophyll family, which includes carotenoid compounds containing oxygen molecules in hydroxyl, epoxy or ketone groups. Astaxanthin is also a metabolite of zeaxanthin and canthaxanthin. It contains several double bonds, which results in a region of decentralized electrons that can help scavenge reactive oxygen species (ROS). Therefore, astaxanthin is a very powerful antioxidant and anti-inflammatory agent [15,16,17]. Astaxanthin is produced naturally by microalgae *Haematococcus pluvialis* and yeast *Xanthophyllomyces dendrorhous* [18], especially when faced by a stressful environment, such as starvation or high salinity. Astaxanthin is also present in animals who feed on the algae, such as salmon, trout, sea bream, flamingos, and crustaceans. The safety of astaxanthin as a dietary supplement intended for humans and animals has been proven [18,19]. However, the effects of astaxanthin on blue light LED-induced retinal damage have not been reported. In the present study, we used 661W photoreceptor cells as a retinal cell model system to investigate the potential protective effects of astaxanthin on blue light-induced retinal injury. Furthermore, we aimed to elucidate the detailed mechanisms underlying these protective effects.

## 2. Results

### 2.1. Astaxanthin Prevented Blue Light LED-Induced 661W Cell Death

**Figure 1:**
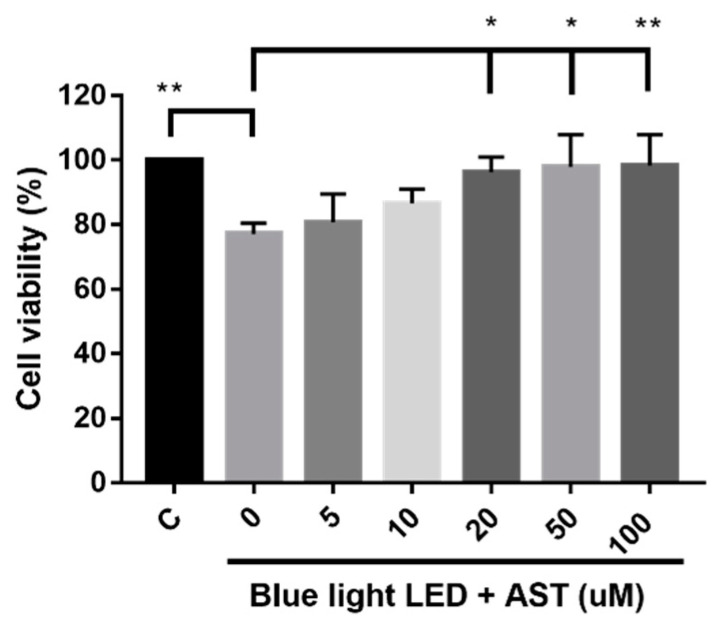
Astaxanthin (AST) suppressed blue light light-emitting diode (LED)-induced cell death. Cell viability was analyzed by Cell Counting Kit-8 (CCK-8) assays. Cells were exposed to 2000 lx blue light LED for 24 h after treatment with various concentrations of AST for 1 h. (C: control group, no AST or blue light LED exposure. All data represent mean ± SD. *p < 0.05, **p < 0.01 compared to the AST-untreated blue light LED exposed group; ANOVA with Dunnett’s multiple comparisons test; N = 5 per group).

The viability of 661W cells, determined using Cell Counting Kit-8 (CCK-8) assay, decreased significantly after exposure to blue light LED, which validated the blue light LED-induced retinal cell damage. Cell viability was significantly higher in the high-dose astaxanthin treatment group compared to that in untreated cells (Figure 1). Moreover, astaxanthin treatment reduced cell death in a dose-dependent manner. Thus, the protective effects of astaxanthin on blue light LED-induced 661W cell death was concentration dependent.

### 2.2. Astaxanthin Inhibited Blue Light LED-Induced 661W Cell Apoptosis 

**Figure 2:**
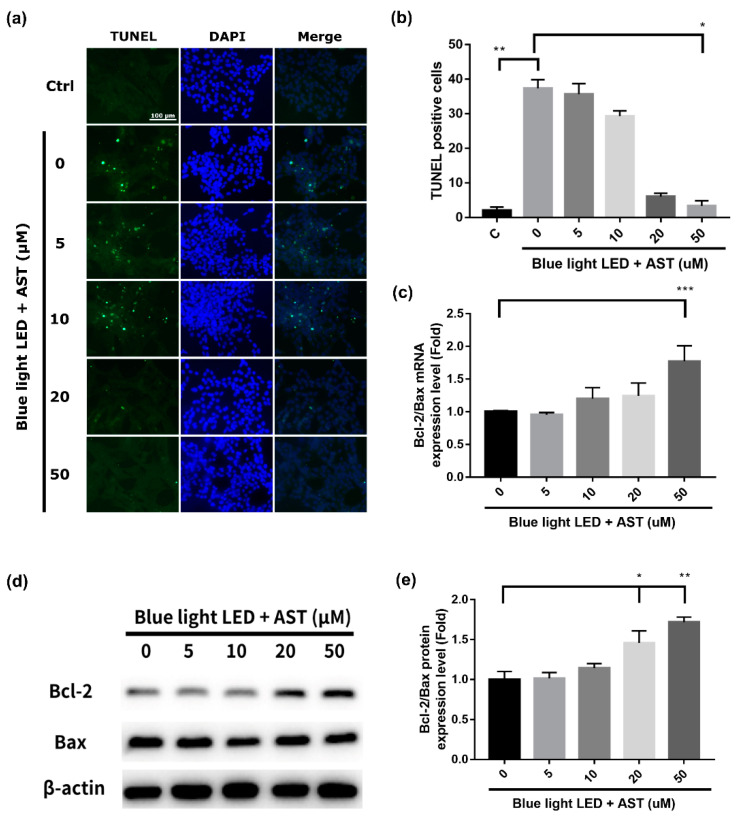
Astaxanthin (AST) inhibited blue light light-emitting diode (LED)-induced 661W cell apoptosis. The 661W cells were exposed to 2000 lx blue light LED for 24 h after being treated with various concentrations of AST for 1 h. (**a**) Apoptotic cells detected by terminal deoxynucleotidyl transferase-mediated dUTP-biotinide end labeling (TUNEL). Nuclei were counterstained with 4′,6-diamidino-2-phenylindole (DAPI). Ctrl: control group—no AST or blue light LED exposure. (**b**) Number of TUNEL-positive cells were counted in at least four randomly chosen views, represented as columns. (C: control group. All data represent mean ± SD. *p < 0.05, **p < 0.01 compared to the AST-untreated blue light LED exposed group; Kruskal–Wallis test with post hoc Dunn test; N = 3 in each group.) (**c**) Evaluation of the relative mRNA expression of B-cell lymphoma 2 (Bcl-2)/Bcl-2-associated X protein (Bax) by quantitative reverse transcription polymerase chain reaction (qRT-PCR). (All data represent mean ± SD. *** *p* < 0.001 compared to the AST-untreated blue light LED exposed group by ANOVA with Dunnett’s multiple comparisons test; N = 5 in each group.) (**d**) Evaluation of the protein expression of Bcl-2 and Bax by Western blot analysis. β-actin was used as the internal control. (**e**) Relative protein expression of Bcl-2/Bax. (All data represent mean ± SD. * *p* < 0.05, ** *p* < 0.01 compared to the AST-untreated blue light LED exposed group by Kruskal–Wallis test with post hoc Dunn test; N = 4 per group.)

We analyzed the damage in 661W cells due to 2000 lx blue light LED exposure using the Terminal deoxynucleotidyl transferase-mediated dUTP-biotinide end labeling (TUNEL) assay. The number of apoptotic cells increased after blue light LED exposure (Figure 2a,b). However, the number of TUNEL-positive cells decreased markedly after astaxanthin pretreatment, especially at higher concentrations (20 and 50 μM). The protective effect was found to be concentration dependent. B-cell lymphoma 2-associated X protein (Bax) is a pro-apoptotic protein, while B-cell lymphoma 2 (Bcl-2) is an important anti-apoptotic protein. We found that astaxanthin pretreatment enhanced the expression of Bcl-2 and inhibited that of Bax; it dose-dependently increased the mRNA and protein expression of Bcl-2/Bax (Figure 2c,d).

### 2.3. Astaxanthin Inhibited ROS Production in 661W Cells

**Figure 3:**
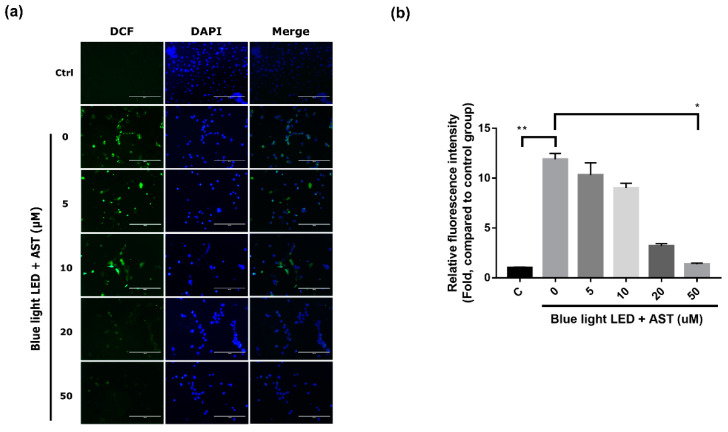
Astaxanthin (AST) inhibited the production of reactive oxygen species (ROS). (**a**) ROS production was analyzed in cells pretreated with different AST concentrations for 1 h after exposure to 2000 lx blue light light-emitting diode (LED) for 24 h. The left column shows 2′, 7′-dichlorfluorescein (DCF) fluorescent cells. Nuclei were counterstained with 4′,6-diamidino-2-phenylindole (DAPI). (Ctrl: control group—no AST or blue light LED exposure.) (**b**) Relative DCF fluorescence intensity in AST pretreated groups compared to that in the control group. (C: control group. All data represent mean ± SD. * *p* < 0.05, ** *p* < 0.01 compared to the AST-untreated blue light LED exposed group; Kruskal–Wallis test with post hoc Dunn test; N = 3 in each group.).

To detect the anti-oxidative effects of astaxanthin on 661W cells, we used an ROS assay to assess ROS production. ROS production increased substantially after exposure to blue light LED. However, astaxanthin pretreatment before exposure led to a significant decline in ROS levels, especially at higher concentrations (20 and 50 μM) (Figure 3). The anti-oxidative effect was also concentration dependent.

### 2.4. Astaxanthin Inhibited Oxidative Stress Biomarkers in 661W Cells 

**Figure 4:**
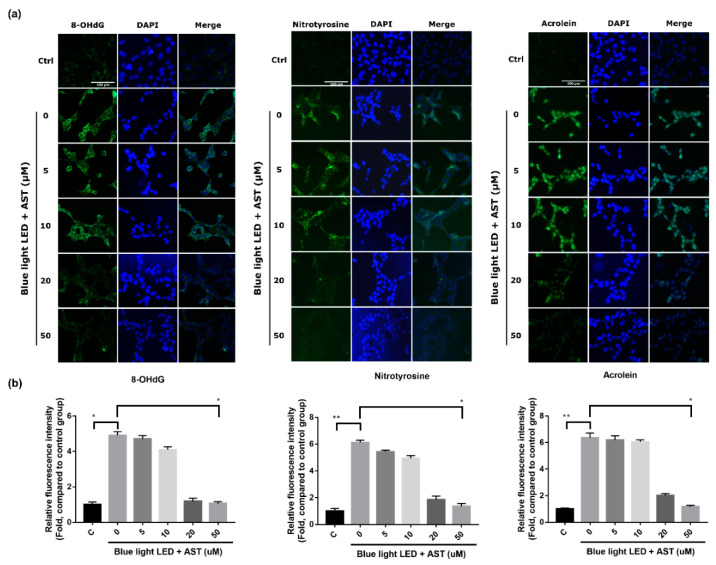
Astaxanthin (AST) inhibited the expression of oxidative stress biomarkers. (**a**) Immunofluorescence detection of 8-hydroxy-2′-deoxyguanosine (8-OHdG), nitrotyrosine, and acrolein in cells pretreated with different AST concentrations for 1 h and then exposed to 2000 lx blue light light-emitting diode (LED) for 24 h. Nuclei were counterstained with 4′,6-diamidino-2-phenylindole (DAPI). (Ctrl: control group—no AST or blue light LED exposure.) (**b**) Relative fluorescence intensity of 8-OHdG, nitrotyrosine, and acrolein compared to that in the control group. (C: Control group. All data represent mean ± SD. * *p* < 0.05, ** *p* < 0.01 compared to the AST-untreated blue light LED exposed group; Kruskal–Wallis test with post hoc Dunn test; N = 3 in each group.)

The expression of 8-hydroxy-2′-deoxyguanosine (8-OHdG), nitrotyrosine, and acrolein, which are indicators of oxidative stress induced by the peroxidation of DNA, protein, and lipid, respectively, increased considerably in 661W cells after exposure to blue light LED. However, pretreatment with astaxanthin before exposure resulted in a significant decrement in fluorescence intensity, especially at higher concentration (20 and 50 μM) (Figure 4). These effects were concentration dependent.

### 2.5. Astaxanthin Suppressed Blue Light LED-Induced Mitochondrial Damage in 661W Cells

**Figure 5:**
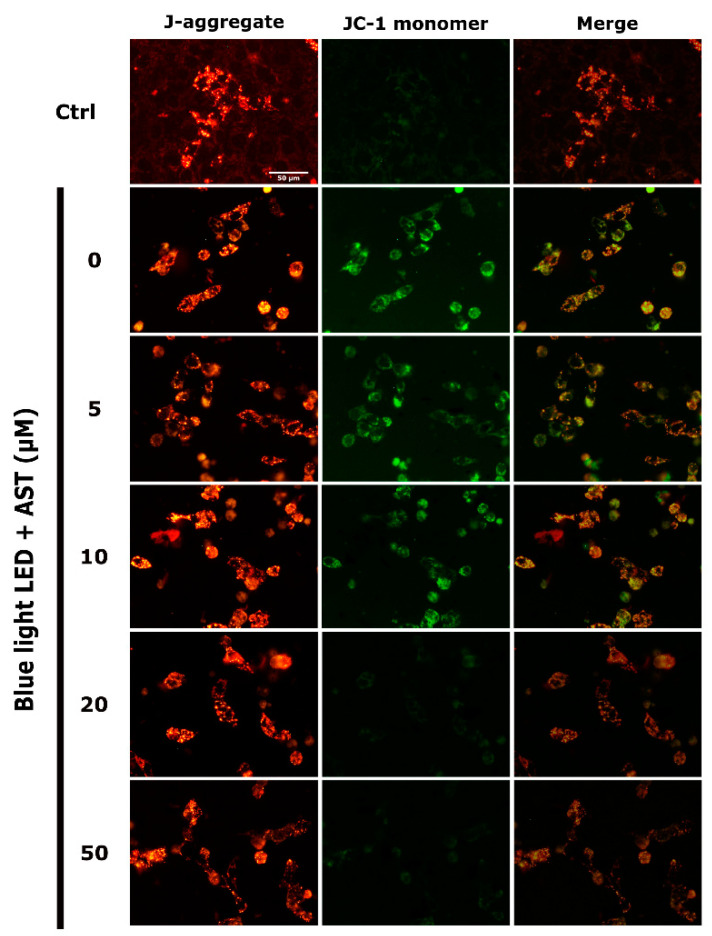
Astaxanthin (AST) protected 661W cells from mitochondrial damage. The 661W cells were subjected to JC-1 staining, which was analyzed by florescence microscopy. J-aggregates and JC-1 monomers were detected using Texas Red and FITC, respectively. Treatment with astaxanthin facilitated JC-1 aggregation and decreased JC-1 monomers in 661W cells exposed to 2000 lx blue light light-emitting diode (LED) for 24 h. (Ctrl: control group, no AST or blue light LED exposure.)

The extent of mitochondrial damage was analyzed by Mitochondrial Membrane Potential Assay Kit (JC-1 method). JC-1 formed J-aggregates in healthy cells and preserved monomeric form in unhealthy or apoptotic cells. Exposure to blue light LED markedly increased JC-1 monomers in 661W cells. However, astaxanthin treatment decreased mitochondrial damage and a higher portion of JC-1 aggregation was observed after blue light LED exposure (Figure 5). Further, the extent of JC-1 aggregation correlated with astaxanthin concentration. 

### 2.6. Astaxanthin Induced Phase II Antioxidant Enzyme Expression and Activated PI3K/Akt/Nrf2 Pathway in 661W Cells

**Figure 6:**
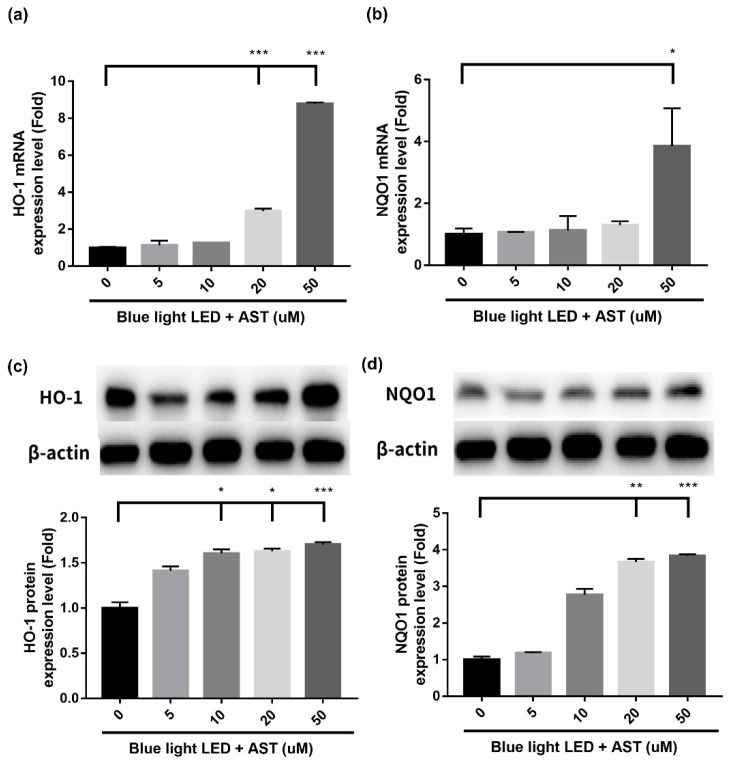
Astaxanthin (AST) upregulated mRNA and protein expression of Phase II enzymes. The 661W cells, pretreated with different AST concentrations for 1 h, were exposed to 2000 lx blue light light-emitting diode (LED) for 24 h. Relative expression of (**a**) heme oxygenase-1 (HO-1); (**b**) NAD(P)H:quinone oxidoreductase-1 (NQO1). Evaluation of the relative mRNA expression by quantitative reverse transcription polymerase chain reaction (qRT-PCR). (All data represent mean ± SD. * *p* < 0.05, *** *p* < 0.001 compared to the AST-untreated blue light LED exposed group by ANOVA with Dunnett’s multiple comparisons test; N = 5 in each group.) Relative protein expression of (**c**) HO-1 and (**d**) NQO1 by Western blot analysis. β-actin was used as the internal control. (All data represent mean ± SD. * *p* < 0.05, ** *p* < 0.01, *** *p* < 0.001 compared to the AST-untreated blue light LED exposed group by Kruskal–Wallis test with post hoc Dunn test; N = 4 per group.)

**Figure 7:**
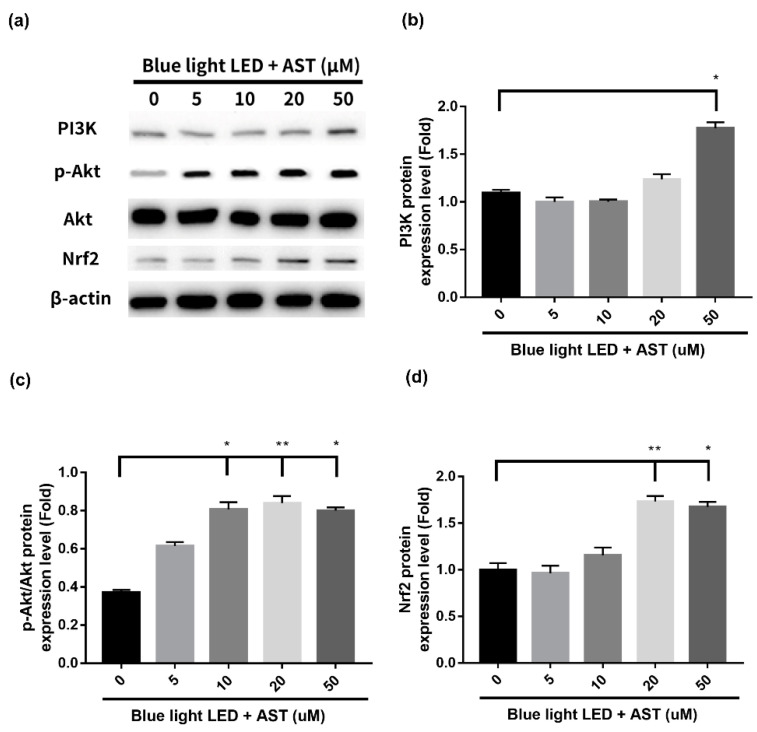
Astaxanthin (AST) activated phosphoinositide 3-kinases (PI3K)/Akt/Nuclear factor erythroid 2-related factor 2 (Nrf2) pathway. The 661W cells, pretreated with different AST concentrations for 1 h, with were exposed to 2000 lx blue light light-emitting diode (LED) for 24 h. (**a**) Evaluation of the protein expression of PI3K, Akt, and Nrf2 by Western blot analysis. β-actin was used as the internal control. (**b**) Relative protein expression of (b) PI3K, (**c**) Phospho-Akt (p-Akt)/Akt, and (**d**) Nrf2. (All data represent mean ± SD. * *p* < 0.05, ** *p* < 0.01 compared to the AST-untreated blue light LED exposed group by Kruskal–Wallis test with post hoc Dunn test; N = 4 per group.)

The present study demonstrated that astaxanthin had anti-oxidative effect against blue light LED-induced 661W cell damage. In order to elucidate the underlying mechanisms of detoxification effect, we examined both the mRNA profiles and protein expression of the factors involved. Phase II enzymes have a detoxification effect against oxidative stress. Treatment with astaxanthin induced an increment in mRNA and protein expression of Heme oxygenase-1 (HO-1) and NAD(P)H:quinone oxidoreductase-1 (NQO1) (Figure 6), which was significant at higher concentrations (20 and 50 μM). Treatment with astaxanthin also induced the protein expression of phosphoinositide 3-kinases (PI3K), Phospho-Akt (p-Akt), and Nuclear factor erythroid 2-related factor 2 (Nrf2) (Figure 7) in a dose-dependent manner. Consequently, we demonstrated that astaxanthin pretreatment activated the PI3K/Akt/Nrf2 pathway and subsequently induced the expression of phase II enzymes in 661W cells.

### 2.7. Astaxanthin-Induced Nuclear Translocation of Nrf2 in 661W Cells

**Figure 8:**
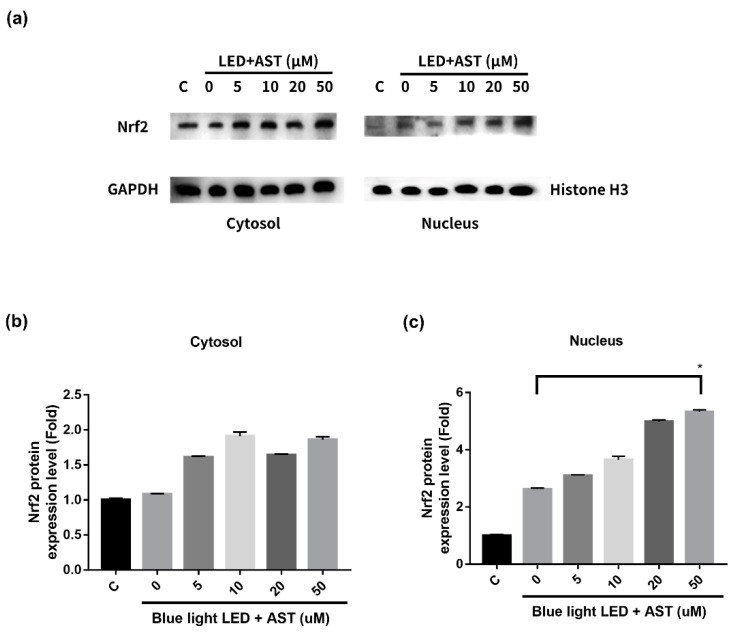
Astaxanthin (AST) induced the nuclear translocation of nuclear factor erythroid 2-related factor 2 (Nrf2) in 661W cells. The 661W cells were exposed to 2000 lx blue light light-emitting diode (LED) for 24 h after treatment with different concentrations of AST for 1 h. (**a**) Evaluation of the protein expression of Nrf2 in cytosol and nucleus by Western blot analysis. Glyceraldehyde-3-phosphate dehydrogenase (GAPDH) and histone H3 were used as the internal controls in cytosol and nucleus, respectively. (**b**) Relative protein expression of Nrf2 in (b) cytosol and (**c**) nucleus. (All data represent mean ± SD. C: control group, no AST or blue light LED exposure. * *p* < 0.05 compared to the AST-untreated blue light LED exposed group by Kruskal–Wallis test with post hoc Dunn test; N = 3 per group.)

The results of Western blot showed that astaxanthin induced the expression of Nrf2 and phase II enzymes. In order to elucidate the modulating mechanisms of Nrf2 activation, we isolated and evaluated the expression of nuclear and cytosolic Nrf2 proteins. When astaxanthin treated 661W cells were exposed to blue light LED, Nrf2 translocated from the cytoplasm to the nucleus (Figure 8). The expression of nuclear Nrf2 increased with astaxanthin concentration. We demonstrated that astaxanthin induced nuclear translocation of Nrf2, which then activated the expression of phase II antioxidant enzymes in 661W cells.

## 3. Discussion

We demonstrated that astaxanthin had a protective effect on 661W photoreceptor cells against damage mediated by blue light LED exposure. Astaxanthin reduced the production of ROS and other oxidative stress biomarkers as well as stabilized the mitochondrial membrane potential of cells. Furthermore, it up-regulated Nrf2-regulated phase II enzymes (HO-1 and NQO1) through the activation of PI3K/Akt pathway. Subsequently, it increased the expression of anti-apoptotic protein Bcl-2, thereby inhibiting cell apoptosis and promoting cell survival. A previous study demonstrated that astaxanthin analogs reduce white fluorescent light-induced photoreceptor degeneration [20]. However, blue light LEDs have higher energy emission and therefore can induce more damage to the retina. To the best of our knowledge the present study is the first to investigate the protective effects of astaxanthin on blue light hazards.

Oxidative stress has been implicated in the pathogenesis of diabetic, hypertensive and ischemic proliferative retinopathy [21]. Previous studies have shown the potential benefits of astaxanthin in retinopathy associated with oxidative stress [22,23,24,25,26]. A strong correlation between oxidative stress and light-induced retinal damage has been previously reported [6,27]. The production of ROS causes tissue injury via the peroxidation of DNA, lipids, proteins, and carbohydrates [28]. Therefore, we detected an increase in the expression of oxidative biomarkers, such as 8-OHdG, nitrotyrosine, and acrolein, in 661W cells after blue light exposure. Oxidative stress also induces mitochondrial dysfunction, and eventually decreases cell viability [29]. Astaxanthin is a strong free radical scavenger and its antioxidant properties are attributed to the physical and chemical interactions with cell membranes. The conjugated double bonds in the polyene chain of astaxanthin trap and scavenge radicals in the cell membrane, quench singlet oxygen, and terminate free radical chain reactions [30]. Taken together, our results demonstrated that astaxanthin attenuated ROS production, decreased mitochondrial damage, and reduced the generation of oxidation products, including 8-OHdG, nitrotyrosine, and acrolein, subsequently enhancing 661W cell survival.

In addition to validating the antioxidant properties of astaxanthin, our study demonstrated that astaxanthin upregulated the Nrf2-antioxidant response element (ARE) pathway and facilitated the expression of phase II antioxidant enzymes, HO-1 and NQO-1. The Nrf2–ARE pathway is an important mechanism to suppress oxidative stress [31]. This mechanism also reportedly protected RPE cells against oxidative damage [32,33]. In the absence of oxidative stress, Nrf2 is bound to the chaperone Kelch-like ECH-associated protein 1 (KEAP1) and is localized in the cytosol. However, in an oxidant environment, the structure of KEAP1 changes and Nrf2 is released, which then translocates to the nucleus and binds to the ARE promoter. Phase II antioxidant enzymes, such as HO-1, and NQO1, are subsequently activated to neutralize oxidative stress [34]. The activation of Nrf2–ARE pathway by astaxanthin was reported in previous studies [35,36,37]. Using RT-PCR and Western blot analysis, our study showed that astaxanthin induced the expression of Nrf2, HO-1, and NQO1 in a dose-dependent manner. ERK, and PI3K/Akt are the two possible mechanisms involved upstream of the Nrf2–ARE pathway [38]. Moreover, both can be activated by astaxanthin [39,40]. However, in previous studies, the PI3K/Akt pathway was found to play important roles in modulating Nrf2–ARE–phase II antioxidant enzymes in response to oxidative stress in RPE cells [33,41]. Although we used 661W cells instead of ARPE-19 cells as our model of blue light LED-induced retinal injury, we observed that astaxanthin induced the expression of PI3K and the phosphorylation of Akt in a dose-dependent manner. In summary, our results indicated that astaxanthin activated the PI3K/Akt pathway, induced the nuclear translocation of Nrf2, increased the expression of phase II antioxidant enzymes, and eventually protected 661W cells against blue light LED-induced oxidative stress.

Astaxanthin has two hydroxyl groups that can span the bi-lipid layer and link the inside and outside of the cell membrane [30]. Moreover, as it has both lipophilic and hydrophilic properties and is fat-soluble, it could be carried by fat molecules to multiple organs and tissues, such as the brain, muscle, and retina [42]. It also has better biological activity than other antioxidants [43] and no toxic effects have been reported. In fact, the United States Food and Drug Administration has approved the use of astaxanthin as a dietary supplement since 1999 [44]. As a potent antioxidant and anti-inflammatory agent, astaxanthin has anti-cancer, anti-diabetic, and immunomodulatory activity [30] as wells as provides protective effects in cardiovascular and neurodegenerative diseases [38,45]. Although astaxanthin is not a component of human retina, it could cross the blood–retinal barrier and exerts anti-oxidative effects on retinal ganglion cells [46].

There were a few limitations in our study. First, our study was conducted in vitro. The protective effects of astaxanthin against blue light damage need to be demonstrated in animal models. Second, the intensity of light exposure in our study was much higher than the typical human daily exposure. Blue light-induced retinal damage is a gradual process and the protective effects of nutrient supplements is difficult to determine. Last but not the least, the blue light-absorbing properties of astaxanthin may also play some roles in its protective effects. However, the results of this study provided strong evidence that astaxanthin may be useful for the prevention of blue light LED-induced retinal injury. Moreover, we proved that the protective effects were mainly attributed to the free radical scavenging activity of astaxanthin and the activation of the PI3K/Akt/Nrf2 pathway.

## 4. Materials and Methods

### 4.1. 661W Cell Culture

The 661W cell line was obtained from Dr. M. Al-Ubaidi (University of Houston). It is a mouse photoreceptor cell line immortalized by the expression of simian virus 40 T antigen, which displays biochemical features of cone cells [47]. The 661W cells were maintained in Dulbecco’s modified Eagle’s media (DMEM) supplemented with 10% phosphate buffer solution (PBS), 100 units/mL penicillin, and 100 μg/mL streptomycin in a 5% CO_2_ atmosphere at 37 °C. Cells were passaged by trypsinization every 3–4 days. Cells from the second to fifth passages were used in the subsequent experiments.

### 4.2. Blue Light LED and Astaxanthin Treatment of 661W Cells

A blue light LED tube (wavelength 450 ± 20 nm, 20 W) was placed at the bottom of a special framework, and we used an illuminometer to measure and conform the light intensity at the cell surface. The distance between the light tube and cell surface was adjusted according to light intensity. The 661W cells seeded on a 96-well plate received LED light exposure directly to minimize the screening effect of astaxanthin in the medium. To ensure a stable environment for 661W cells, the device was placed in the CO_2_ incubator. Temperature was maintained at 36.5–37.5 °C. Astaxanthin (average molecular weight 596.84) was purchased from Sigma-Aldrich (CAS Number 472-61-7, St. Louis, MO, USA). Cells were treated with astaxanthin at various concentrations (0, 5, 10, 20, 50, 100 μM) for 1 h and then exposed to blue light LED with an intensity of 2000 lx for 24 h. Cells were cultured for 24–48 h after exposure. Astaxanthin remained in the medium during the LED exposure.

### 4.3. CCK-8 Assay for Cell Viability

The viability of astaxanthin-treated and blue light LED exposed cells was determined using the CCK-8 assay (TEN-CCK8, Biotools, New Taipei City, Taiwan), according to manufacturer’s instructions. The 661W cells were cultured in 96-well plates and then incubated for 24 h in a 5% CO_2_ atmosphere at 37 °C. The absorbance was measured at 450 nm with a microplate reader (Bio-Rad Laboratories, CA, USA).

### 4.4. TUNEL Assay

TUNEL assay was performed by a FragEL^TM^ DNA fragmentation detection kit (Calbiochem, Darmstadt, Germany). The TUNEL enzyme and peroxidase converter were added to the cells after incubation for 5 min in a permeabilizing solution containing 0.1% Triton-X and 0.1% sodium citrate. After adding FITC–Avidin, image analysis was used to detect the fluorescent signals of the TUNEL–positive cells in four random slides per sample.

### 4.5. Detection of Intracellular ROS

Intracellular ROS levels were quantified using 2′,7′-dichlorodihydrofluorescein diacetate (2′,7′-DCFDA; Sigma-Aldrich, MO, USA) oxidation. To astaxanthin pretreated and blue light LED exposed cells, 10 mM 5-(and-6)-chloromethyl-2′,7′-DCFDA, acetyl ester (CM-H_2_DCFDA), a free radical probe, was added and incubated for 1 h at 37 °C. The radical probe was converted to 2′,7′-dichlorodihydrofluorescein (DCFH) by intracellular esterase. Intracellular DCFH (non-fluorescent) was oxidized to 2′,7′-dichlorfluorescein (DCF, fluorescent) by intracellular ROS. Fluorescence was measured by a microplate reader (Bio-Rad Laboratories, CA, USA) at 485 nm (excitation) and 535 nm (emission).

### 4.6. Immunofluorescence (IF) Detection of Oxidative Stress Biomarkers

IF was performed by blocking and permeabilizing the cells with 0.2% Triton in PBS containing 5% goat serum for 1 h at 25 °C, then incubating them with primary antibodies diluted in blocking solution overnight at 4 °C, and finally incubating with the appropriate fluorescent-labelled secondary antibodies (diluted 1:1000 in blocking solution; Santa Cruz, TX, USA) for 3 h at 25 °C. Nuclei were counterstained with 4′,6-diamidino-2-phenylindole (DAPI). Primary antibodies included mouse anti-rat 8-OHdG (JaICA, Shizuoka, Japan), mouse anti-rat nitrotyrosine (Abcam, Hong Kong, China), and mouse anti-rat acrolein (Abcam, Hong Kong, China). Fluorescence was measured by a microplate reader (Bio-Rad Laboratories, CA, USA).

### 4.7. Determination of Mitochondrial Dysfunction

The mitochondrial membrane potential of cells was analyzed by the JC-1 Mitochondrial Membrane Potential Assay Kit (Cayman Chemical Company, MI, USA). After 24 h of 2000 lx blue LED light exposure, 50 µL of JC-1 staining buffer was added to 1 mL of culture medium. The fluorescence signals for both J-aggregates with Texas Red (healthy cells, excitation/emission = 560/595 nm) and JC-1 monomers with FITC (apoptotic or unhealthy cells, excitation/emission = 485/535 nm) were captured by a fluorescence microscope. The number of cells (red or green stained cells) were counted in a blinded manner by Image-J.

### 4.8. RNA Extraction and Quantitative Reverse Transcription Polymerase Chain Reaction

RNA was extracted from 661W cells by TRIzol reagent (Invitrogen-Life Technologies, MD, USA). For each sample, 1 μg of total RNA was incubated with 300 ng oligo(dT) (Promega, WI, USA) for 5 min at 65 °C and reverse transcribed into cDNA using 80 U of Moloney murine leukemia virus reverse transcriptase (MMLV-RT; Thermo Fisher Scientific, MA, USA) per 50 μg of reaction sample for 1 h at 37 °C. The reaction was terminated by heating the samples at 90 °C for 5 min. The resultant cDNA product was subjected to PCR with specific primers. The primer sequences were as follows: HO-1: 5′-AAGATTGCCCAGAAAGCCCTGGAC-3′ (forward), 5′-AACTGTCGCCACCAGAAAGCTGAG-3′ (reverse); NQO1: 5′-AAGGATGGAAGAAACGCCTGGAGA-3′ (forward), 5′-GGCCCACAGAAAGGCCAAATTTCT-3′ (reverse); Bcl-2: 5′-CTGGTGGACAACATCGCTCTG-3′ (forward), 5′-GGTCTGCTGACCTCACTTGTG-3′ (reverse); Bax: 5′-TGGTTGCCCTTTTCTACTTTG-3′ (forward), 5′-GAAGTAGGAAAGGAGGCCATC-3′ (reverse); Glyceraldehyde-3-phosphate dehydrogenase (GAPDH): 5′-ACCACAGTCCATGCCATCAC-3′ (forward), 5′-TCCACCACCCTGTTGCTGTA-3′ (reverse). The 50 μL reaction mixture contained 5 μL cDNA, 1 μL sense and antisense primers each, 200 μM of each deoxynucleotide, 5 μL of 10× Taq polymerase buffer, and 1.25 U Taq polymerase (Promega, WI, USA). A thermocycler (MJ Research, MA, USA) was used for amplification with the following conditions: Initial 1 min denaturation at 94 °C and 3 min extension at 72 °C. The annealing temperature between 42 °C and 62 °C was decreased by 1 °C decrements, which was followed by 21 cycles at 55 °C. Finally, the temperature was elevated to 72 °C for 10 min and then reduced to 4 °C. The products were subjected to electrophoresis on a 2% agarose gel and analyzed by a gel analyzer system. Each mRNA level was standardized based on the intensity of GAPDH.

### 4.9. Western Blot Analysis

661W cells were pretreated with different concentrations of astaxanthin for 1 h, followed by exposure to 2000 lx blue light LED for 24 h. Then, the cells were washed with PBS, lysed in RIPA buffer (Sigma-Aldrich, MO, USA) containing 1% protease inhibitor cocktail and 1% phosphatase inhibitor cocktails 2 and 3 (Sigma-Aldrich, MO, USA), and harvested. Lysates were centrifuged at 12,000 *g* for 15 min at 4 °C. Protein concentrations were quantified by a BCA Protein Assay Kit (Bio-Rad Laboratories, CA, USA) with bovine serum albumin as the standard. The protein samples were separated by 10% SDS-PAGE gradient electrophoresis and then transferred to polyvinylidene difluoride membranes (Immobilon-P; Millipore, MA, USA). The primary antibodies utilized in the experiment were as follows: rabbit anti- HO-1 (1:2500, Abcam, Hong Kong, China), mouse anti- NQO1 (1:2000, Abcam, Hong Kong, China), rabbit anti- PI3K (1:1000, Cell Signaling Technology, MA, USA), rabbit anti-Akt (1:1000, Cell Signaling Technology, MA, USA), rabbit anti- p-Akt (1:1000, Cell Signaling Technology, MA, USA), rabbit anti-Bax (1:1000, Cell Signaling Technology, MA, USA), rabbit anti- Bcl-2 (1:500, Proteintech, IL, USA), rabbit anti-Nrf2 (1:500, Abcam, Hong Kong, China), and mouse anti-β-actin (1:5000, Sigma-Aldrich, MO, USA). A horseradish peroxidase (HRP)-conjugated goat anti-rabbit antibody and an HRP-conjugated goat anti-mouse antibody were used as secondary antibodies. Immunodetection was performed by enhanced chemiluminescence (Pierce Biotechnology, MA, USA). Protein levels were measured using densitometry analysis of the bands. β-actin was used as an internal control.

### 4.10. Nuclear and Cytosolic Protein Extraction and Analysis of Nrf2 Protein Expression

Nuclear and cytosolic proteins were extracted separately using NE-PER (Nuclear and Cytoplasmic Extraction Reagent; Pierce Biotechnology, MA, USA) according to the manufacturer’s instructions. Proteins were quantified by a BCA Protein Assay Kit (Bio-Rad Laboratories, CA, USA) and Western blot was performed with anti-Nrf2 antibody (1:500, Abcam, Hong Kong, China). GAPDH (1:5000, Abcam, Hong Kong, China) was used as the internal control in cytosol and histone H3 (1:1000, Cell Signaling Technology, MA, USA) was used as the internal control in nucleus.

### 4.11. Statistical Analyses

Data are presented as mean ± SD. To compare numerical data among the groups, one-way ANOVA or Kruskal–Wallis test, followed by post hoc test was used. *p* < 0.05 was considered statistically significant. Statistical analysis was performed by SPSS software (version 17.0, SPSS Inc., Chicago, IL, USA).

## 5. Conclusions

Our study was the first effort to investigate and demonstrate the protective effects of astaxanthin on retinal photoreceptor cells against damage mediated by blue light LED exposure. Astaxanthin upregulated the PI3K/Akt/Nrf2-ARE signaling pathway and subsequently activated the expression of phase II antioxidant enzymes (HO-1 and NQO1). As a strong free radical scavenger, it also reduced the production of ROS, up-regulated the anti-apoptotic protein Bcl-2, and stabilized the mitochondrial membrane. Thus, this study supported the idea that astaxanthin could be potentially used to prevent blue light-induced retinal damage.
